# A dahlia flower extract has antidiabetic properties by improving insulin function in the brain

**DOI:** 10.1093/lifemeta/load026

**Published:** 2023-06-18

**Authors:** Dominik Pretz, Philip M Heyward, Jeremy Krebs, Joel Gruchot, Charles Barter, Pat Silcock, Nerida Downes, Mohammed Zubair Rizwan, Alisa Boucsein, Julia Bender, Elaine J Burgess, Geke Aline Boer, Pramuk Keerthisinghe, Nigel B Perry, Alexander Tups

**Affiliations:** Centre for Neuroendocrinology, School of Biomedical Sciences, University of Otago, Dunedin 9054, New Zealand; Department of Physiology, School of Biomedical Sciences, University of Otago, Dunedin 9054, New Zealand; Maurice Wilkins Centre for Molecular Biodiscovery, University of Auckland, Auckland 1010, New Zealand; Department of Physiology, School of Biomedical Sciences, University of Otago, Dunedin 9054, New Zealand; Maurice Wilkins Centre for Molecular Biodiscovery, University of Auckland, Auckland 1010, New Zealand; Department of Medicine, University of Otago, Wellington, Wellington South 6242, New Zealand; Centre for Endocrine Diabetes and Obesity Research, Wellington Regional Hospital, Newtown, Wellington 6021, New Zealand; Centre for Neuroendocrinology, School of Biomedical Sciences, University of Otago, Dunedin 9054, New Zealand; Department of Physiology, School of Biomedical Sciences, University of Otago, Dunedin 9054, New Zealand; Department of Medicine, University of Otago, Wellington, Wellington South 6242, New Zealand; Product Development Research Centre, University of Otago, Dunedin 9054, New Zealand; Product Development Research Centre, University of Otago, Dunedin 9054, New Zealand; Centre for Neuroendocrinology, School of Biomedical Sciences, University of Otago, Dunedin 9054, New Zealand; Department of Physiology, School of Biomedical Sciences, University of Otago, Dunedin 9054, New Zealand; Maurice Wilkins Centre for Molecular Biodiscovery, University of Auckland, Auckland 1010, New Zealand; Centre for Neuroendocrinology, School of Biomedical Sciences, University of Otago, Dunedin 9054, New Zealand; Department of Physiology, School of Biomedical Sciences, University of Otago, Dunedin 9054, New Zealand; Maurice Wilkins Centre for Molecular Biodiscovery, University of Auckland, Auckland 1010, New Zealand; Centre for Neuroendocrinology, School of Biomedical Sciences, University of Otago, Dunedin 9054, New Zealand; Department of Chemistry, The New Zealand Institute for Plant and Food Research, University of Otago, Dunedin 9054, New Zealand; Centre for Neuroendocrinology, School of Biomedical Sciences, University of Otago, Dunedin 9054, New Zealand; Department of Physiology, School of Biomedical Sciences, University of Otago, Dunedin 9054, New Zealand; Maurice Wilkins Centre for Molecular Biodiscovery, University of Auckland, Auckland 1010, New Zealand; Centre for Neuroendocrinology, School of Biomedical Sciences, University of Otago, Dunedin 9054, New Zealand; Department of Physiology, School of Biomedical Sciences, University of Otago, Dunedin 9054, New Zealand; Department of Chemistry, The New Zealand Institute for Plant and Food Research, University of Otago, Dunedin 9054, New Zealand; Centre for Neuroendocrinology, School of Biomedical Sciences, University of Otago, Dunedin 9054, New Zealand; Department of Physiology, School of Biomedical Sciences, University of Otago, Dunedin 9054, New Zealand; Maurice Wilkins Centre for Molecular Biodiscovery, University of Auckland, Auckland 1010, New Zealand

**Keywords:** inflammation, hypothalamus, signal transduction, neuroendocrine, arcuate nucleus

## Abstract

Butein, a rare chalcone found in the toxic plant *Toxicodendron vernicifluum*, has been shown to regulate glucose homeostasis via inhibition of the nuclear factor kappa-B kinase subunit beta (IKKβ)/nuclear factor kappa B (NF-κB) pathway in the brain. Here, we investigated whether the nonpoisonous plant *Dahlia pinnata* could be a source of butein as a potential treatment for type 2 diabetes (T2D). In mice fed a high-fat diet (HFD) to induce glucose intolerance, an oral *D. pinnata* petal extract improved glucose tolerance at doses of 3.3 mg/kg body weight and 10 mg/kg body weight. Surprisingly, this effect was not mediated by butein alone but by butein combined with the closely related flavonoids, sulfuretin and/or isoliquiritigenin. Mechanistically, the extract improved systemic insulin tolerance. Inhibition of phosphatidylinositol 3-kinase to block insulin signaling in the brain abrogated the glucoregulatory effect of the orally administered extract. The extract reinstated central insulin signaling and normalized astrogliosis in the hypothalamus of HFD-fed mice. Using NF-κB reporter zebrafish to determine IKKβ/NF-κB activity, a potent anti-inflammatory action of the extract was found. A randomized controlled crossover clinical trial on participants with prediabetes or T2D confirmed the safety and efficacy of the extract in humans. In conclusion, we identified an extract from the flower petals of *D. pinnata* as a novel treatment option for T2D, potentially targeting the central regulation of glucose homeostasis as a root cause of the disease.

## Introduction

Type 2 diabetes (T2D) is a leading cause of death and an increasingly serious threat to global health. Worldwide, the number of people suffering from T2D has increased 4-fold since 1980 and is predicted to increase to 552 million by 2030 [1]. Poorly managed T2D leads to decreased quality of life and shorter life expectancy. Serious complications of T2D include kidney failure, cardiovascular disease, and blindness, and the cost of treating diabetes accounts for 12% of global health expenditure [2].

In 1855, Claude Bernard made the remarkable discovery that the brain can control blood glucose [3], but it took almost 150 years before this finding was revisited. It is now widely accepted that glucose homeostasis is tightly regulated by central pathways mainly residing in the hypothalamus [4–7]. In a healthy state, circulating insulin reaches the hypothalamus and binds to its receptor, which is then autophosphorylated. The autophosphorylation of the receptor leads to the recruitment and phosphorylation of the insulin receptor substrate (IRS). This induces the activation of the phosphatidylinositol 3-kinase (PI3K)—protein kinase B (AKT) pathway, which mediates most of the metabolic effects of insulin [8, 9].

Glucose intolerance and insulin resistance are key features in the pathogenesis of T2D. Hypothalamic inflammation has been shown to play a key role in the development and progression of hypothalamic insulin resistance through inhibition of the nuclear factor kappa-B kinase subunit beta (IKKβ)/nuclear factor kappa B (NF-κB) inflammatory pathway [10, 11]. NF-κB is a transcription factor that regulates the expression of proinflammatory cytokines such as interleukin (IL)-1 and tumor necrosis factor-­alpha (TNF-α). We and others have shown that inhibition of this pathway in neurons of the arcuate nucleus (ARC) in the hypothalamus attenuates glucose intolerance in diet-induced obese (DIO) and glucose-intolerant mice [10–12]. Pharmacological inhibition of this pathway using the potent and specific IKKβ inhibitor butein, administered either centrally or systemically, had marked glucose-lowering and insulin-sensitizing effects in diet-induced obese and glucose-intolerant mice [10].

These results suggested that a nontoxic, natural source of butein could be a novel treatment that targets hypothalamic inflammation as a root cause of T2D. However, yellow chalcone butein is a rather rare flavonoid [13]. One of the best described natural sources of butein is the bark of the Chinese lacquer tree (*Toxicodendron vernicifluum*). Although this plant has been used in traditional herbal medicine to treat a range of conditions, such as inflammatory diseases, its medicinal use has been limited because of its toxicity [14]. Interestingly, the yellow petals of the ornamental flower varieties of nontoxic *Dahlia pinnata* Cav. (Asteraceae) have also been shown to contain butein [15]. This current study aimed to establish whether an extract of the yellow petals of *D. pinnata* could be a novel therapy for the treatment of T2D from a cultivatable source. We found that acute and chronic oral treatment with this extract improved glucose tolerance in high-fat diet (HFD) fed mice. A closer examination of the extract revealed that, even though butein was found in high concentrations in the extract, it was only effective in combination with either one or two other flavonoids, sulfuretin and/or isoliquiritigenin, which were also isolated from the dahlia extract. We established that the glucoregulatory effects of the extract were dependent on PI3K signaling as a marker of central insulin action and that it reverses markers of hypothalamic inflammation. In a “first in human” clinical study on the dahlia extract in people with prediabetes and T2D, we confirmed its promising glucose-lowering action.

## Results

### Dahlia extract improves glucose tolerance and insulin sensitivity in DIO mice

**Figure 1:**
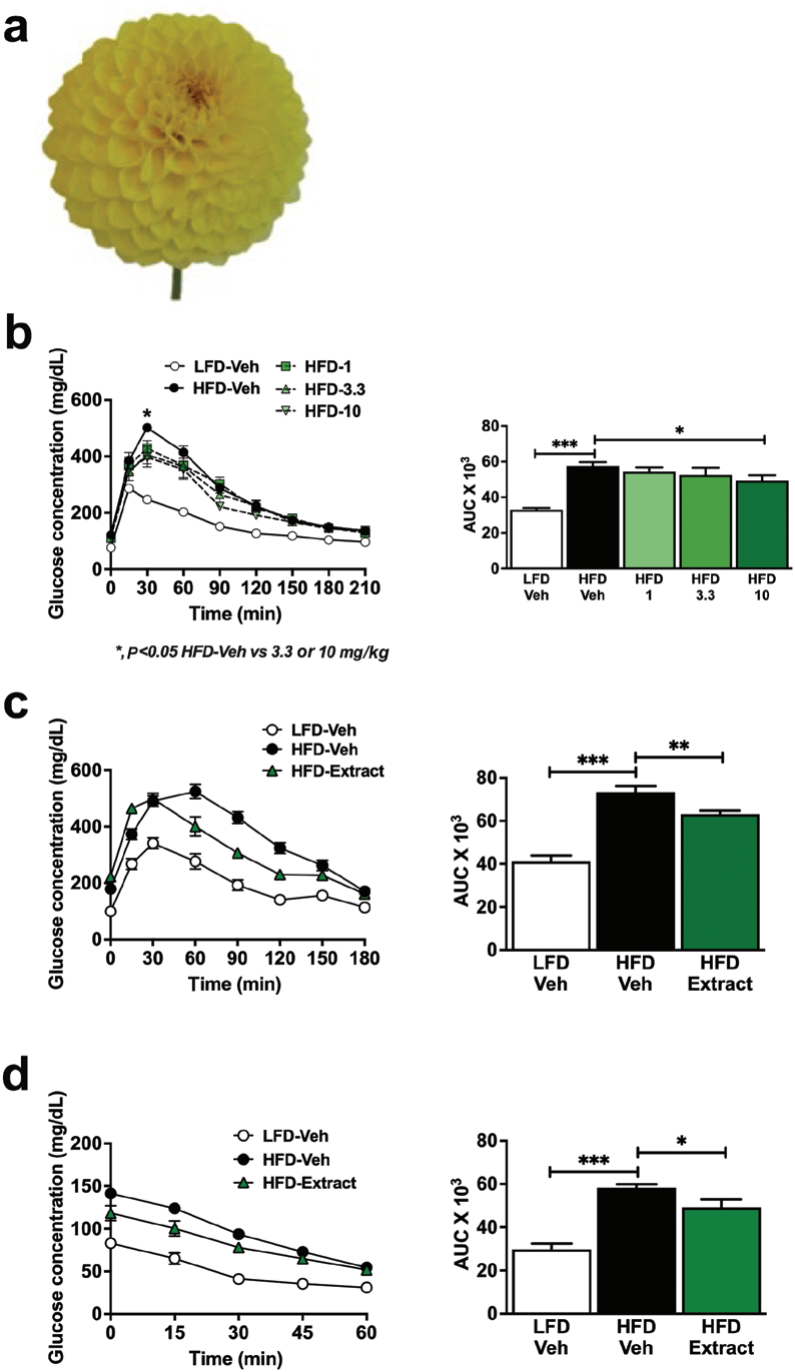
The dahlia extract improved glucose and insulin tolerance in DIO mice. (a) *Dahlia pinnata* flower as the source of the dahlia extract. C57BL/6 mice were fed HFD or LFD for 4 weeks and treated with the extract orally (1, 3.3, or 10 mg/kg) 1 h before an ipGTT or ITT. (b) ipGTT blood glucose response curve (left panel) and corresponding AUC (right panel; *n* = 6–7). C57BL/6 mice were fed the HFD or the LFD for 4 weeks and followed by oral administration of either dahlia extract (10 mg/kg body weight) or vehicle before ipGTT or ITT. (c) ipGTT blood glucose response curve (left panel) and corresponding AUC (right panel; *n* = 6–7). (d) ITT blood glucose response curve (left panel) and corresponding AUC (right panel; *n* = 7–8). Data are represented as the mean ± SEM. ^*^P ≤ 0.05, ^**^P ≤ 0.01, ^***^P ≤ 0.001.

HPLC analysis on an ethanol (EtOH) extract of fresh yellow petals of *D. pinnata* (flower shown in Fig. 1a) showed a high concentration of butein (Supplementary Tables S1 and S2). To test whether the dahlia extract could improve glucose tolerance, we next tested the effect of different doses of the extract in HFD-fed mice. This is a well-established animal model for the study of glucoregulation [16–18]. Doses of 1, 3.3, and 10 mg/kg body weight were administered orally 1 h before an intraperitoneal glucose tolerance test (ipGTT). The extract improved glucose tolerance, with 10 mg/kg body weight being the most effective dose, as reflected by the area under the curve (AUC) (*P* < 0.05, Fig. 1b). Thirty minutes after the ipGTT glucose load, there were significant decreases in glucose levels compared with the HFD vehicle group, at extract doses of 3.3 mg/kg body weight (*P* = 0.02) and 10 mg/kg body weight (*P* = 0.04) (Fig. 1b). To demonstrate the reproducibility of the effect, we repeated the experiment using the 10 mg/kg body weight dahlia extract dose and tested its effects on glucose and insulin tolerance (ITT). Oral application of the dahlia extract (10 mg/kg body weight) to HFD-fed mice consistently improved glucose tolerance (*P* = 0.008; Fig. 1c), confirming its effectiveness, and this improvement was associated with an increase in insulin sensitivity (*P* = 0.04; Fig. 1d). The extract did not affect blood sugar levels in healthy control mice fed a low-fat diet (LFD) (Supplementary Fig. S1).

### Dahlia extract improves glucose homeostasis after long-term administration in mice

To test sustained glucoregulation and possible adverse effects of long-term treatment with the dahlia extract, we treated a cohort of HFD-fed mice daily with oral dahlia extract (10 mg/kg body weight) or vehicle for 5 weeks.

**Figure 2:**
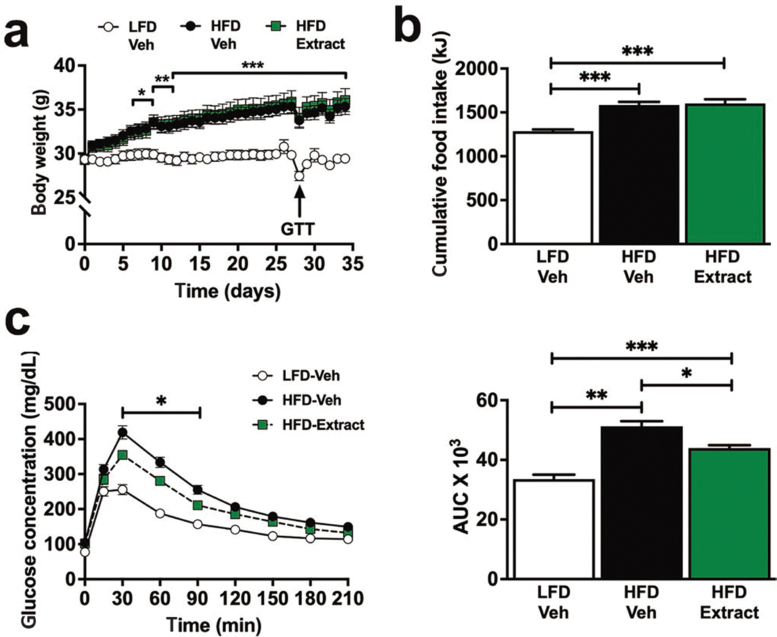
The dahlia extract remained effective after long-term treatment. C57BL/6 mice were fed the HFD or the LFD *ad libitum* and treated with the extract (10 mg/kg/d) or vehicle by oral gavage once a day for 4 weeks. (a) Body weight (*n* = 10). Indicated significance refers to the difference between the HFD Vehicle (Veh) or HFD Extract treatment group and the LFD Veh treatment group. (b) Cumulative food intake for each treatment group (*n* = 10). (c) GTT blood glucose response curve (left panel) and corresponding AUC (right panel) (*n* = 7–10). Data are represented as the mean ± SEM. ^*^P ≤ 0.05, ^**^P ≤ 0.01, ^***^P ≤ 0.001.

HFD feeding led to an increase in body weight from 29.3 ± 0.5 g to 35.3 ± 0.9 g over 5 weeks (*P* < 0.001 compared with mice fed the LFD, Fig. 2a). Chronic administration of the dahlia extract did not alter the body weight of mice fed the HFD (HFD 35.3 ± 0.9 g vs HFD extract 36.1 ± 1.2 g). Mice fed the HFD showed increased cumulative food intake compared with mice fed the LFD (1584 ± 117 kJ vs 1285 ± 20 kJ, *P* < 0.0001), and this was unaffected by daily treatment with the extract (Fig. 2b). An ipGTT performed after 4 weeks of HFD feeding showed that HFD feeding led to impaired glucose tolerance compared with mice fed the LFD (*P* < 0.001, Fig. 2c). HFD-fed mice that received long-term treatment with the extract showed improved glucose tolerance compared with mice fed the HFD that received vehicle (*P* = 0.02, Fig. 2c).

To assess toxicity, we measured total liver weight and liver fat content and assessed liver morphology. Chronic daily administration of the dahlia extract did not alter liver morphology, liver fat content, and total liver weight (Supplementary Fig. S2). This proved the safety of the dahlia extract at the tested dose in mice over a prolonged treatment period.

### Butein, isoliquiritigenin, and sulfuretin are the bioactives in the dahlia extract that mediate the glucose-lowering effect in certain combinations

**Figure 3:**
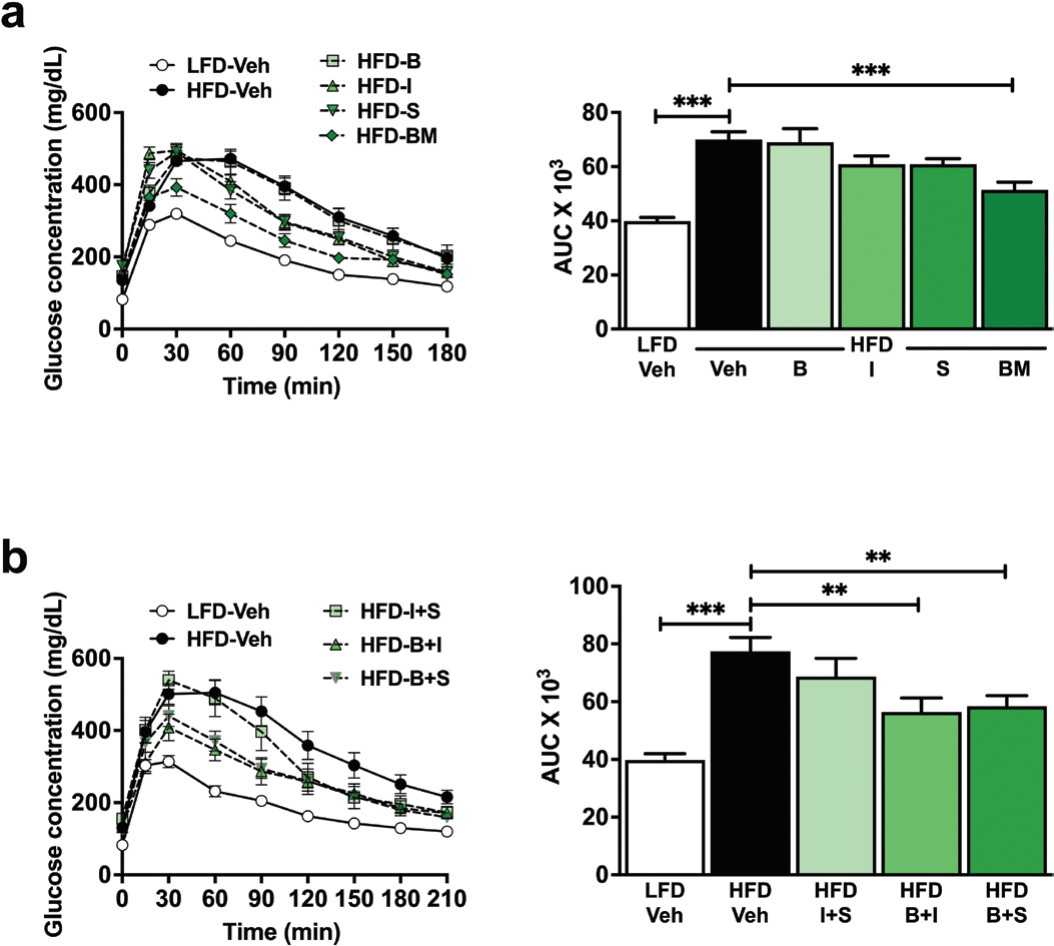
Yellow dahlia flavonoids show glucose-lowering effects in combination. C57BL/6 mice were fed the HFD or the LFD for 4 weeks, and flavonoids were administered orally individually or in combination 1 h before an ipGTT. Shown are ipGTT blood glucose response curves (left panels) and corresponding AUCs (right panels). (a) Mice received butein (B, 10 mg/kg), isoliquiritigenin (I, 10 mg/kg), sulfuretin (S, 10 mg/kg), or a mixture of all three (BM, 3.3 mg/kg each); (*n* = 10–30). (b) Mice received isoliquiritigenin+sulfuretin (I + S), butein+isoliquiritigenin (B+I), or butein+sulfuretin (B+S), 3.3 mg/kg for each flavonoid (*n* = 6–17). Data are represented as the mean ± SEM. ^*^P ≤ 0.05, ^**^P ≤ 0.01, ^***^P ≤ 0.001.

We next sought to identify the bioactives that elicit the glucoregulatory effects of the dahlia extract. Our group previously showed that pure butein improved glucose homeostasis through intracerebroventricular (ICV) administration in DIO mice or when administered orally to obese, leptin-deficient mice [10]. Intriguingly, oral butein did not improve glucose homeostasis in DIO mice in the current study (Fig. 3a).

This finding led us to revisit our HPLC analysis, and we found that the dahlia extract contained two other yellow flavonoids besides butein. These flavonoids were purified and identified as the chalcone isoliquiritigenin and the aurone sulfuretin (Supplementary Fig. S3, Supplementary Tables S1 and S2). Neither isoliquiritigenin nor sulfuretin alone (10 mg/kg body weight) significantly improved glucose tolerance in HFD-fed mice that exhibited impaired glucose tolerance (Fig. 3a). However, an equimolar mixture of all three flavonoids, referred to as a bioactive mixture (BM; 3.3 mg/kg body weight of each flavonoid), profoundly improved glucose tolerance (*P* = 0.001; Fig. 3a).

We next tested whether any combination of two of these three flavonoids improves glucose tolerance. Combinations of butein with isoliquiritigenin (AUC; *P* = 0.004) or sulfuretin (AUC; *P* = 0.005) significantly improved glucose tolerance, whereas the combination of isoliquiritigenin and sulfuretin (AUC; *P* = 0.445) was ineffective (Fig. 3b). Therefore, we can conclude that butein is required in combination with one or both of the other yellow flavonoids to elicit the glucoregulatory effect.

### The glucoregulatory effect of the dahlia extract depends on PI3K, a marker of central insulin signaling, and the extract reverses diet-induced hypothalamic inflammation

**Figure 4:**
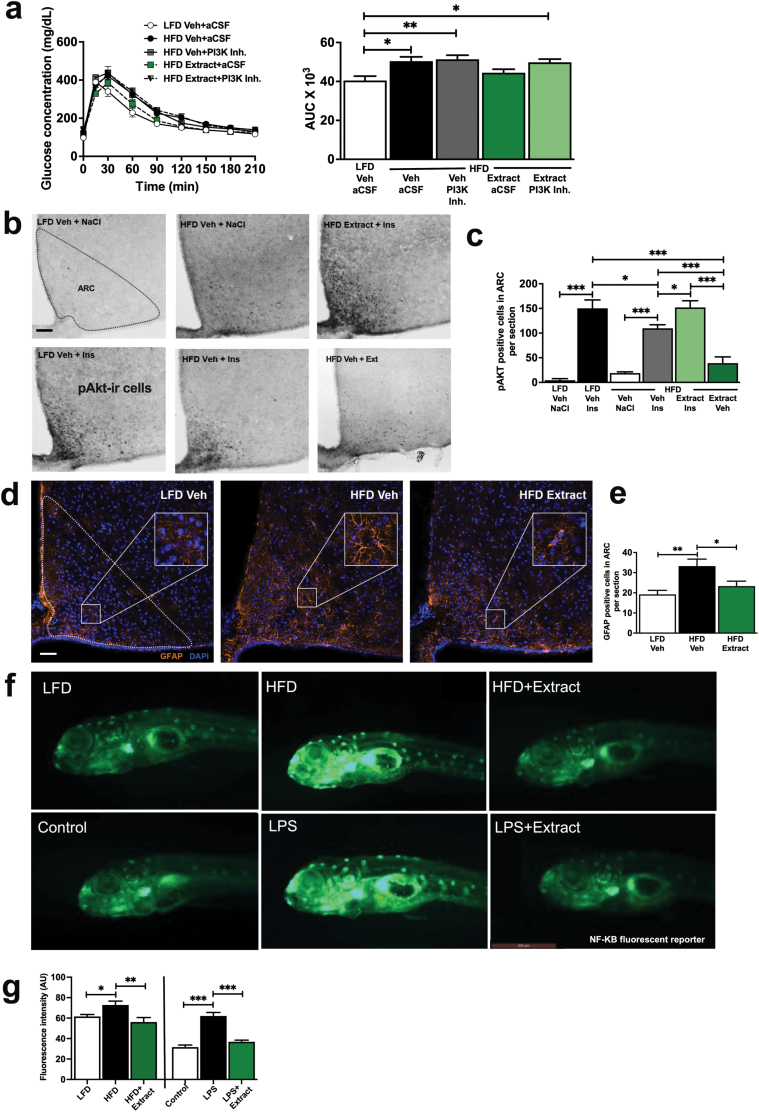
The dahlia extract sensitizes central insulin signaling, reduces astrogliosis, and blocks the IKKβ/NFκB pathway, and the glucose-lowering effects of the extract are abolished by blockade of central insulin signaling. (a) Central action of the extract. C57BL/6 mice were fed the HFD or the LFD for 4 weeks and received an ICV injection of a PI3K inhibitor cocktail or vehicle 1 h before the GTT. The extract (10 mg/kg) was given orally 60 min before the GTT. Left panel: GTT. Right panel: AUC of (a) (*n* = 6–7) (b) pAKT-positive cells in the ARC (framed area), indicating insulin responsiveness. C57BL/6 mice were fed the HFD or the LFD for 4 weeks and were treated orally with the extract (10 mg/kg) or the bioactive mixture (3.3 mg/kg of each flavonoid) 1 h before fixative perfusion. Mice also received an intraperitoneal injection of insulin (1 mg/kg) or vehicle 15 min before perfusion. (c) Quantification of pAKT-positive cells. pAKT immunoreactive cells were counted within three region-matched sections for each animal (*n* = 3–7). (d) GFAP immunoreactive cells within the ARC (framed areas), indicative of the astrocyte population. C57BL/6 mice were fed the HFD or the LFD and treated with the extract (10 mg/kg) by oral gavage once a day for 5 weeks. (e) Quantification of GFAP immunoreactive cells. GFAP immunoreactive cells were counted in three region-matched sections for each animal (*n* = 8–10). Data show means ± SEM. ^*^P≤ 0.05, ^**^P ≤ 0.01. (f and g) Anti-inflammatory action of the extract. NF-κB reporter zebrafish were either fed with an LFD, fed with egg yolk to induce HFD conditions, or treated with LPS (10 µmol/L) for 4 h followed by incubation with the extract (10 µmol/L) for one additional hour (*n* = 8 per group). (f) Representative images of fish of the treatment groups. (g) NF-κB activity was measured by quantitative analysis of fluorescence intensity. Data are represented as the mean ± SEM. ^*^P ≤ 0.05, ^**^P ≤ 0.01, ^***^P ≤ 0.001.

Having established that the dahlia extract exhibited glucose-­lowering effects in mice, we next investigated if this was a centrally mediated effect. Since the central insulin signaling pathway is crucial for the regulation of blood glucose levels [6–8, 19, 20], we assessed the effect of the dahlia extract on glucose tolerance mediated via this pathway. Central insulin signaling was inhibited by selective inhibitors of the PI3K catalytic subunits, p110α and p110β (PIK75 and TGX221), that mediate insulin signaling in the brain [9]. Treating HFD-fed mice with the dahlia extract restored their glucoregulation to the level of LFD-fed controls (*P* = 0.55, Fig. 4a). But when insulin signaling was blocked by ICV injection of PI3K inhibitors, the glucose-lowering effect of the dahlia extract was abolished, and glucose tolerance in mice fed the HFD remained significantly impaired compared with mice fed the LFD (*P* = 0.007, Fig. 4a), showing a central mode of action of the dahlia extract.

The ARC of the hypothalamus is an essential brain region for the regulation of glucose homeostasis, so we tested whether the dahlia extract affected insulin signaling in this region. Mice were fed the LFD or the HFD for 5 weeks. During this period, the HFD-fed mice were treated daily with dahlia extract (10 mg/kg body weight) or vehicle by oral gavage. At baseline without insulin treatment, mice fed the LFD or the HFD had few pAKT-positive cells in the ARC (Fig. 4b and c). Insulin injection markedly increased the number of pAKT-positive cells in the ARC of mice fed the LFD (35-fold increase, *P* < 0.001), but to a lesser degree in mice fed the HFD (6-fold increase, *P* < 0.001). The increase of pAKT-positive cells upon insulin injection was 37% greater in LFD-fed mice compared to HFD-fed mice (*P* = 0.05). This indicates the presence of central insulin resistance in the latter group. When treated with the dahlia extract, the total number of pAKT-positive cells in mice fed the HFD following insulin injection was restored to levels of the LFD group (*P* = 0.03). The extract did not lead to a significant increase in the number of pAKT cells in the ARC of mice fed HFD in the absence of exogenous insulin. These results suggest normalization of central insulin signaling by the extract (Fig. 4b and c).

Impaired central insulin sensitivity is linked to hypothalamic inflammation [21, 22], therefore, we counted glial fibrillary acidic protein (GFAP)-positive, activated astrocytes, a marker of inflammation, within the ARC of mice fed the HFD following chronic treatment with the dahlia extract or vehicle. The number of GFAP-positive cells in the ARC was markedly increased in vehicle-treated mice fed the HFD compared with vehicle-treated mice fed the LFD (73% increase, *P* = 0.004; Fig. 4d and e). This finding is consistent with previous research [23]. The dahlia extract reversed this effect. Mice fed the HFD treated with the extract showed fewer GFAP-positive astrocytes than mice fed the HFD treated with the vehicle (30% reduction, *P* = 0.05; Fig. 4d and e). This suggests that dahlia extract can reduce hypothalamic inflammation.

Since butein is a specific IKKβ inhibitor [24, 25], we next tested whether the dahlia extract can inhibit activation of the proinflammatory IKKβ/NF-κB pathway. We utilized a zebrafish reporter line that carries a green fluorescent protein (GFP) reporter gene [26], allowing visualization of NF-κB activity, the downstream target of IKKβ. HFD feeding for 5 h at the fifth day post fertilization (dpf) increased fluorescence intensity relative to the control group (*P* < 0.05), as did treatment with the positive control lipopolysaccharide (LPS) (*P* < 0.001; Fig. 4f and g). LPS was used as a positive control as it induces NF-κB activity [27], and the use of LPS showed that this zebrafish model can detect a known NF-κB response. Incubation with the dahlia extract for the last hour of HFD or LPS treatment reversed NF-κB pathway activation to levels observed in the control groups (*P* < 0.001; Fig. 4f and g). These data confirm that the extract inhibits the IKKβ/NF-κB pathway and has marked anti-inflammatory properties.

Taken together, these results suggest that the glucose-­lowering properties of the dahlia extract are mediated via its central anti-inflammatory action, specifically the ability to inhibit the IKKβ/NF-κB pathway.

### Dahlia extract improves glucose tolerance in humans

**Figure 5:**
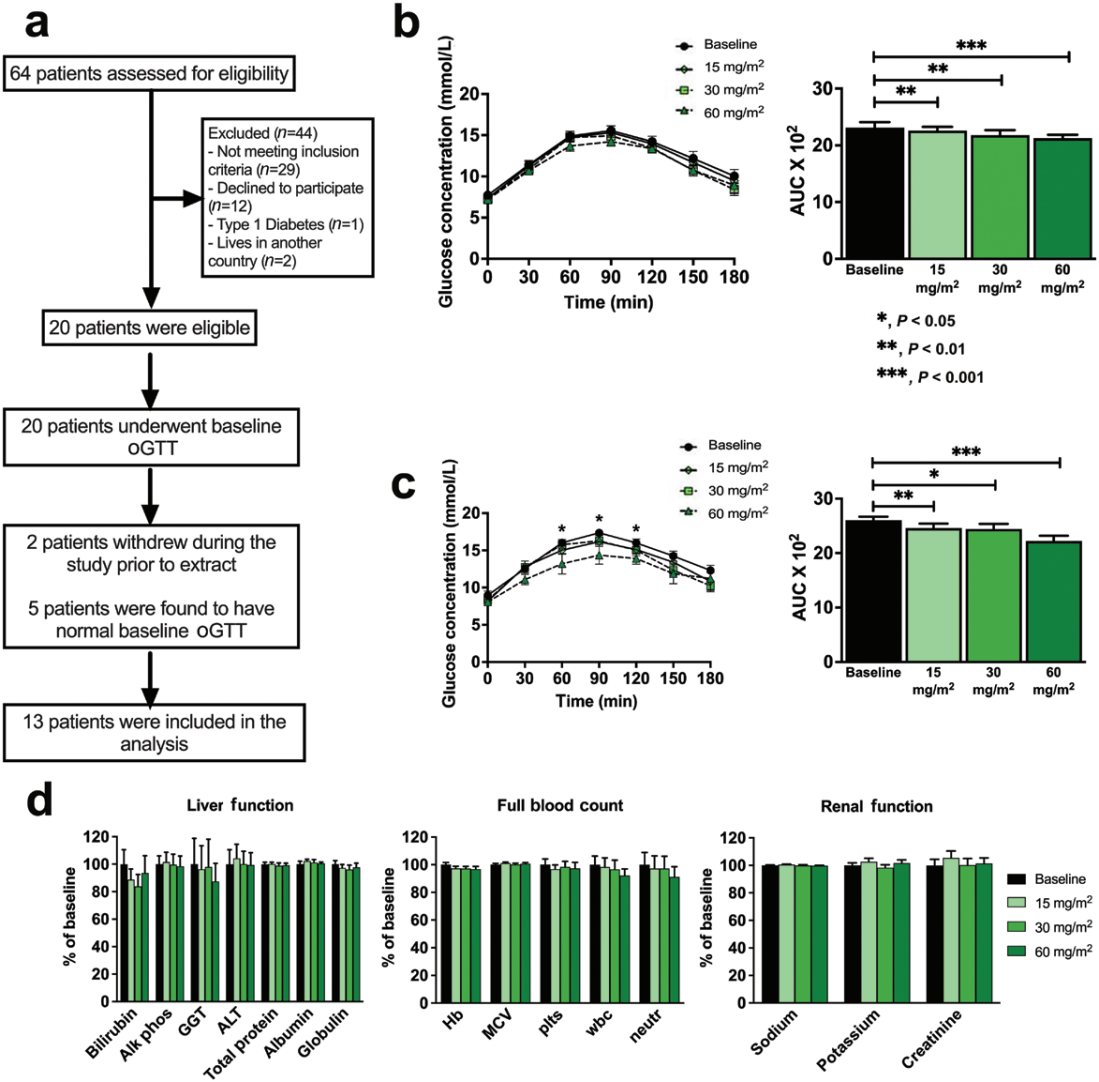
“First in man” clinical trial of the dahlia extract. (a) Study summary flow chart. (b) GTT blood glucose response curves (left panel) and corresponding AUCs (right panels) for all participants with prediabetes or T2D (*n* = 13). The extract (15, 30, or 60 mg/m^2^) was given 1 h before the GTT. (c) GTT and AUC results for participants (*n* = 5) with established T2D (HbA1c >48 mmol/mol). (d) Liver function, full blood count, and renal function were unchanged by treatment with the dahlia extract. Data are represented as the mean ± SEM. ^*^P ≤ 0.05, ^**^P ≤ 0.01, ^***^P ≤ 0.001. Abbreviations: Alb, albumin; ALT, alanine aminotransferase; AP, alkaline phosphatase; Bi, bilirubin; GGT, gamma-glutamyl transpeptidase; Glo, globulin; Hb, hemoglobin; MCV, mean corpuscular volume; neutr, neutrophils; plts, platelets; TP, total protein; WBC, white blood cells.

Having shown the glucose-lowering effects of the dahlia extract and a potential mechanism of action in preclinical animal models, we tested the safety and efficacy of the extract in humans. We conducted a randomized controlled crossover trial of 13 participants with prediabetes or T2D (WHO criteria) [28] who fulfilled the inclusion criteria (Fig. 5a). The mean age of these participants was 55.9 ± 7.2 years, with HbA1c of 46.5 ± 4.5 mmol/mol. The participants’ body weights were 64.2–178.1 kg, and their BMI were 22.8–54.4 kg/m^2^.

The dahlia extract improved glucose tolerance in the patients with prediabetes or T2D, and the dahlia extract showed a dose-dependent improvement in glucose tolerance, as reflected by the AUC during an oral glucose tolerance test (oGTT) for each dose (Fig. 5b; 15 mg/m^2^: *P* = 0.0021, 30 mg/m^2^: *P* = 0.0079, and 60 mg/m^2^: *P* = 0.0002). When examining the only five participants meeting the WHO criteria for established T2D (HbA1c ≥ 48 mmol/mol), the glucose-lowering effect of the highest dahlia extract dose (60 mg/m^2^) was more pronounced (Fig. 5c; 15 mg/m^2^: *P* = 0.0061, 30 mg/m^2^: *P* = 0.0213, and 60 mg/m^2^: *P* = 0.0008), suggesting an increased effect of the dahlia extract in patients who had already progressed to T2D.

Blood parameters that reflected liver function (bilirubin, alkaline phosphatase, gamma-glutamyl transferase, alanine transaminase, total protein, albumin, and globulin), renal function (sodium, potassium, and creatinine), and overall health (hemoglobin, mean corpuscular volume, platelets, white blood cells, and neutrophils) did not differ between pretreatment measurements and those taken during or after any dose of the dahlia extract (Fig. 5d).

## Discussion

An EtOH extract of fresh petals of yellow dahlia contained a high concentration of butein. This extract, administered orally, acutely improved glucose tolerance, and improved insulin sensitivity in DIO mice. Following chronic treatment, the glucose-lowering properties of the dahlia extract were retained. This is important regarding the potential long-term use of dahlia extract to improve and sustain glucoregulation. Dahlia extract had a dose-­dependent glucose-lowering effect in a “first in human” study undertaken in people with prediabetes and T2D, in which a more prominent effect was observed in those diagnosed as having “established” T2D. These are the first results on the antidiabetic properties of dahlia flower extract.

We previously found that oral butein improves glucose tolerance and central insulin signaling in obese and glucose-intolerant mice that are deficient for the body weight and glucoregulatory hormone leptin [10]. While butein was also effective in DIO mice when it was administered ICV [10], the current study revealed that oral application of butein in DIO mice was ineffective. This apparent dependence on the route of butein application may reflect the effect of leptin on intestinal barrier function, altering the absorption rate of specific compounds [29], or differences in blood–brain barrier (BBB) function between HFD-fed and leptin-deficient mice [30].

Since oral administration of the dahlia extract but not purified butein improved glucose tolerance in DIO mice, our data further suggest that the high concentration of butein in the dahlia extract is not solely responsible for mediating the glycemic effects of the extract. We isolated two other yellow flavonoids from the extract, the chalcone isoliquiritigenin and the aurone sulfuretin. Isoliquiritigenin has been shown to decrease adipose tissue inflammation in mice [31] via modifying gut bacteria composition [32], and sulfuretin appears to protect mice against cytokine-induced β-cell damage and prevent streptozotocin-induced diabetes [33]. However, without co-administration of butein, these flavonoids, administered in isolation or combined, did not improve glucose tolerance. These apparent differences may be due to different routes of application, e.g., intraperitoneal injection vs oral gavage application in our study. Even though butein did not exhibit glucose-lowering properties when administered orally in isolation, combinations of butein with isoliquiritigenin and/or sulfuretin did, confirming that butein is essential to elicit the glucose-lowering effect of the dahlia extract. This might be due to functional interactions between the flavonoids or their metabolites *in vivo*. The individual compounds may exhibit different modes of action, e.g., by influencing brain accessibility which needs to be evaluated in future studies. While our current results show that butein, isoliquiritigenin, and sulfuretin are the main bioactives of the dahlia extract, mediating its *in vivo* glucose-­lowering effects, we cannot rule out contributions of other secondary plant metabolites in the dahlia extract.

The flavonoids may be processed by microbiota, intestinal cells, or in the liver before reaching the target tissues. For example, isoliquiritigenin is converted into seven Phase-I metabolites by human liver microsomes *in vitro* [34]. Only small amounts of unmodified sulfuretin were found in the plasma of rats after oral administration of a sulfuretin-rich plant extract, with most of the sulfuretin being present in the form of conjugates [35]. Further studies are needed to investigate whether the beneficial effects on glucose homeostasis induced by the dahlia extract are mediated through the flavonoids present in the extract or by their metabolites.

The brain plays a pivotal role in the regulation of glucose homeostasis, with impaired central insulin signaling considered a primary factor in the development of T2D [36, 37]. Within the brain, the hypothalamus plays a fundamental role in glucoregulation [38]. It was shown that unlike peripheral inflammation, hypothalamic inflammatory signaling and reactive gliosis develop very rapidly in response to HFD feeding before substantial weight gain. While this initial response subsided after several days, by 4 weeks of HFD consumption, inflammation, and reactive gliosis were fully established and considered a permanent characteristic of this state. For these reasons, we investigated the glucoregulatory effects of the extract and the bioactives in this well-established model of DIO [39]. Hypothalamic inflammation and central insulin signaling are tightly linked, and therefore treatments targeting hypothalamic inflammation would address a primary cause of dysregulated glucose metabolism and improve glucose tolerance in the long-term. To act in the hypothalamus, bioactive compounds must cross the BBB, a highly selective semipermeable barrier that separates the cerebrospinal fluid from the bloodstream [40]. Various phytochemicals can traverse the BBB independently of their route of administration [41–46]. Within the brain, phytochemicals are associated with the inhibition of oxidative stress and signaling pathways related to inflammation. For example, epigallocatechin-3-gallate, the major catechin in green tea, induces the nuclear factor erythroid-related factor 2 (Nrf2) pathway in hippocampal neurons, protecting them against oxidative damage [47]. The flavanone hesperetin and two of its metabolites, hesperetin-7-O-β-d-glucuronide and 5-nitro-hesperetin, inhibit oxidative stress-induced apoptosis in cortical neurons via activation of AKT and extracellular signal-regulated kinases 1/2 (ERK1/2). These are also important components of the IRS-AKT pathway involved in glucose homeostasis [48]. Our findings show that hypothalamic insulin signaling is a target of the dahlia extract. At the molecular level, HFD feeding leads to low-grade inflammation in this brain area, contributing to insulin resistance and ultimately to the development of T2D [39]. HFD feeding promptly leads to reactive astrogliosis in the hypothalamus, reflecting the proinflammatory nature of the diet. This is believed to contribute to the functional impairment of neuronal circuits controlling energy homeostasis [39]. The complete loss of effectiveness of the dahlia extract after blocking central insulin signaling suggests that the extract has a central mode of action. Our data further suggest that the dahlia extract prevents the onset of astrogliosis after HFD feeding, and the improvement of glucose tolerance is associated with the suppression of inflammatory signaling pathways within the hypothalamus. Whether the improved insulin sensitivity, as reflected by increased activation of hypothalamic AKT, is a result of decreased inflammation or a direct activation by the flavonoids requires further investigation.

The improvement in glucose homeostasis observed in response to the extract was not accompanied by adverse effects in preclinical models and clinical trial. Hypoglycemia did not occur in any mouse treated with the dahlia extract. Chronic daily administration of the dahlia extract did not alter liver morphology, liver fat content, or liver weight. Furthermore, in zebrafish, the dahlia extract reduced hyperactivity of NF-κB induced by HFD feeding or LPS, but did not reduce NF-κB activity to levels below controls. Many poisonous compounds affect body weight and food intake [49, 50], but the chronic treatment of mice with the extract did not reveal differences in these parameters. This supports the safety of the dahlia extract at the tested doses over a prolonged treatment period. Evidence for the absence of toxicity in humans is provided by the safety parameter analysis of the blood obtained from the clinical study. No differences were detected after administration of the dahlia extract in liver and renal function. Results of full blood count were identical between different treatments.

In summary, we have shown that a yellow dahlia flower extract, or its isolated yellow flavonoids in certain combinations, can restore glucoregulation in mice fed HFD to induce glucose intolerance. In humans, the dahlia extract improved glucoregulation in people with prediabetes and T2D. The glucoregulatory effect of the extract is dependent on central PI3K, a key component in insulin signaling. The improvement in glucose tolerance is associated with an anti-inflammatory effect of the dahlia extract and increased insulin signaling in the hypothalamus, the major glucoregulatory region of the brain.

## Materials and methods

### Flower extracts and flavonoids

Yellow dahlia flowers from a cultivar of *D. pinnata* Cav. (synonym *D. variabilis* Desf., detailed information can be acquired via the website of The World Flora Online) were grown in Southland (46° South, New Zealand). Extracts and flavonoids were prepared from these flowers as detailed in the Supplemental Material. HPLC analyses showed the flavonoid composition (Supplementary Table S1). HPLC analysis further confirmed that butein, sulfuretin, and isoliquiritigenin were stable in an EtOH extract of the dahlia petals when stored at −18°C for at least 3 years and in the capsules stored for at least 6 months. For mouse trials that utilized flavonoids and flavonoid mixtures, commercial samples of butein (Sigma Aldrich, purity 98%) and isoliquiritigenin (AK Scientific, purity 97%) were used. Sulfuretin was purified from the dahlia extract as it was not commercially available (Supplemental Material, purity >98% by HPLC and ^1^H NMR analyses).

### Mice

For all mouse experiments, 12–14 weeks old male C57BL/6 mice were obtained from the University of Otago (UoO) animal facility. Mice were housed individually under a 12 h light/12 h dark cycle at 23°C. To induce DIO, mice were fed an HFD (60% fat; Cat. No. D12492; Research Diets). Mice fed an LFD (10% fat; Cat. No. D12450B; Research Diets) served as controls.

### ipGTT

After 4 weeks of either HFD or LFD feeding, mice were fasted overnight for 16 h and received dahlia extract (1, 3.3, or 10 mg/kg body weight) or vehicle (0.9% NaCl containing 5% EtOH) by oral gavage. Glucose (1.5 g/kg body weight; dissolved in 0.9% NaCl) was injected intraperitoneally, and an ipGTT was performed 60 min after dahlia extract or vehicle administration. Blood glucose levels were measured using a commercially available glucometer (Accu-Check Performa; Roche) after truncating the tip of the tail with a fresh scalpel blade. The AUC was calculated. In a separate cohort of mice, an ipGTT was performed 60 min after oral gavage with either 10 mg/kg butein, sulfuretin, isoliquiritigenin, or combinations of each flavonoid (3.3. mg/kg of each flavonoid).

### ITT

After 4 weeks of either HFD or LFD feeding, mice were fasted overnight for 16 h and received dahlia extract (10 mg/kg body weight) or vehicle through oral administration. Insulin (Sigma Aldrich, 1 mg/kg body weight; dissolved in 0.9% NaCl) was injected intraperitoneally 60 min after oral gavage with the dahlia extract or vehicle, and blood glucose measurements were performed as described above.

### Chronic treatment studies

Mice were fed the HFD or the LFD *ad libitum* for 5 weeks and received daily dahlia extract (10 mg/kg body weight, oral gavage) or vehicle (0.9% NaCl containing 5% EtOH) treatment. Body weight and food intake were measured daily. After 4 weeks of treatment, mice were subjected to an ipGTT as described above. At the end of the treatment period, mice were sacrificed, and their livers were weighed. Liver morphology was assessed as described in [51].

### ICV infusion of PI3K inhibitors

Mice were fed the HFD or the LFD for 2 weeks, after which ICV surgeries were performed under isoflurane anesthesia as described previously [52]. Following surgery, mice were fed the HFD or the LFD for an additional 2 weeks. After a 16 h overnight fast, a PI3K inhibitor cocktail [0.1 nmol in 2% DMSO/artificial spinal fluid (aCSF)], PIK-75 and TGX-221, Sigma Aldrich, both isoforms are required for insulin signaling in the central nervous system (CNS) [9] or vehicle (2% DMSO/aCSF) was infused ICV. After 60 min, the dahlia extract (10 mg/kg body weight) or vehicle (0.9% NaCl containing 5% EtOH) was administered by oral gavage and animals were subjected to an ipGTT 60 min later, as described above.

### Immunohistochemistry

Mice were fed the LFD or the HFD for 5 weeks and treated with the dahlia extract (10 mg/kg body weight) once daily by oral gavage. After 5 weeks, mice were fasted overnight for 16 h. They received a final administration of the extract (10 mg/kg body weight) or vehicle (0.9% NaCl containing 5% EtOH) by oral gavage 60 min before transcardial perfusion. Additionally, they received insulin (1 mg/kg body weight; dissolved in 0.9% NaCl) or vehicle (0.9% NaCl) injections 15 min before transcardial perfusion. Immunohistochemistry was carried out on mouse brain coronal cryosections as described previously [52], using an anti–phospho-AKT Ser473 antibody (Cat. No. 4058; Cell Signaling Technology).

In a different cohort of mice, astrocytes were stained after a long-term treatment to identify potential anti-inflammatory properties in the ARC. Mice were fed the LFD or the HFD for 5 weeks while treated with the extract (10 mg/kg/d) or vehicle (0.9% NaCl containing 5% EtOH) once daily by oral gavage. After 5 weeks, mice were fasted overnight for 16 h and sacrificed by transcardial perfusion. Immunohistochemistry was carried out on mouse brain coronal cryosections as described previously [23], using an anti-GFAP antibody (Cat. No. ab53554; Abcam). Immunoreactive cells within the ARC were counted by two investigators blinded to the treatment.

### Measurement of IKKβ-NF-κB activity in zebrafish

Zebrafish (NF-κB responsive reporter zebrafish, pSGNluc/Tg (8×Hsa.NFκB:GFP, Luciferase) strain) were maintained in 3.5 L tanks on a Palletized Centralized Life Support System (Tecniplast) on a 14 h light:10 h dark cycle. The water was kept at 26–30°C, with pH 7.6–8.0 and a conductivity of 300–600 µS. Only larvae at 5 dpf were used for this study. At 5 dpf, larvae (*n* = 8) were incubated for 6 h in HFD (vortexed chicken egg yolk, 2% final concentration in E3 medium), LPS (10 µmol/L in E3 medium), or vehicle (E3 medium). After 5 h, either dahlia extract (2.75 µg/mL) or vehicle (E3 medium) was added, and fish were incubated for an additional hour before imaging. Images were taken using a Leica M205 FA epifluorescence microscope and analyzed using ImageJ. Activation of the IKKβ-NF-κB pathway was quantified by manually drawing a region of interest around the larva, followed by measurement of average fluorescent intensity in the region of interest as described in Ref. [53].

### Clinical study

A double blind, crossover clinical study in people with prediabetes or T2D was run on 13 male patients aged 18–70 years with HbA1c concentrations 40–65 mmol/mol. Potential participants underwent screening blood tests for HbA1c, full blood count, electrolytes, creatinine, and liver function tests. Exclusion criteria were: glucose-lowering medication, previous bariatric surgery, liver disease with aspartate (AST) or alanine aminotransferase (ALT) greater than three times the upper limit of normal, renal disease with an eGFR <60 mL/min/1.73 m^2^, or known cardiac disease.

Dahlia extract doses were calculated using a standard conversion from mouse results: Human Equivalent Dose (mg/kg) = Animal dose (mg/g) × (Animal K_m_/Human K_m_) [54]. This was then converted for body surface area for the human subjects to adjust for the volume of distribution: 0.007184 × (Weight in kg)^0.425^ x (Height in cm)^0.725^ [55]. The mouse trial beneficial dose of 10 mg/kg was converted to a human dose of 30 mg/m^2^. Additional half and double doses were chosen, i.e., 15 and 60 mg/m^2^.

The study supplementation consisted of capsules containing 5, 20, or 50 mg of powdered dahlia extract. We performed a sequential, random order crossover study. Each participant underwent a baseline standardized 75 g oGTT. After a 12 h overnight fast, participants drank 75 g of anhydrous glucose dissolved in H_2_O at 0 min. Venous blood samples were drawn at 0 min, then every 30 min until 3 h post ingestion. If the fasting plasma glucose was <6.1 mmol/L and the 2 h post-oral glucose load plasma glucose concentration was <7.8 mmol/L, the participant was withdrawn from the study.

At least 72 h after the initial baseline oGTT, participants took one of three doses of dahlia extract, selected in random order, and then underwent another oGTT after 1 h. Both participants and researchers were blinded to the dose and order. Venous blood was taken to measure glucose, C-Peptide, and insulin at 0 min and then every 30 min for 3 h following glucose ingestion. Wash-out periods of 1 week separated subsequent visits, at which the participants underwent the same procedure with the remaining two doses.

Participants were monitored for adverse reactions, with half-hourly observations of heart rate, blood pressure, respiratory rate, oxygen saturation, and temperature during the oGTTs. The following blood parameters were determined the day before and after each oGTT: bilirubin, alkaline phosphatase, gamma-­glutamyl transferase, alanine transaminase, total protein, albumin, globulin for liver function, sodium, potassium, and creatinine for renal function and hemoglobin, mean corpuscular volume, platelets, white blood cells, and neutrophils for a full blood count. The primary outcome of the human study was the AUC of the oGTT over 3 h for the three selected doses of the dahlia extract compared with the AUC of the baseline oGTT. Secondary outcomes included safety monitoring of renal and hepatic function and a full blood count.

### Statistics

The data are presented as means ± SEM. Statistical analysis was conducted using Graphpad Prism software (Graphpad Prism, Version 7, Graphpad Software). Where appropriate, the data were subjected to unpaired one-way ANOVA followed by a Holm-Šidák comparison test, or repeated measures one-way ANOVA, followed by uncorrected Fischer’s LSD test as appropriate. Differences were considered significant if *P* < 0.05.

## Supplementary Material

load026_suppl_Supplementary_Material

## Data Availability

All study data are included in the article and/or supplementary information. Materials and reagents are available upon request.

## References

[1] Saeedi P, Petersohn I, Salpea P et al. IDF Diabetes Atlas Committee. Global and regional diabetes prevalence estimates for 2019 and projections for 2030 and 2045: results from the International Diabetes Federation Diabetes Atlas. Diabetes Res Clin Pract 2019;157:107843. 31518657 10.1016/j.diabres.2019.107843

[2] Williams R, Karuranga S, Malanda B et al. Global and regional estimates and projections of diabetes-related health expenditure: results from the International Diabetes Federation Diabetes Atlas. Diabetes Res Clin Pract 2020;162:108072. 32061820 10.1016/j.diabres.2020.108072

[3] Bernard C. Leçons de physiologie expérimentale appliquée à la médecine: faites au Collège de France. Paris: JB Baillière et fils, 1855–56.

[4] Brüning JC, Gautam D, Burks DJ et al. Role of brain insulin receptor in control of body weight and reproduction. Science (New York, N.Y.) 2000;289:2122–5. 11000114 10.1126/science.289.5487.2122

[5] Mirzadeh Z, Faber CL, Schwartz MW. Central nervous system control of glucose homeostasis: a therapeutic target for type 2 diabetes? Annu Rev Pharmacol Toxicol 2022;62:55–84. 34990204 10.1146/annurev-pharmtox-052220-010446 PMC8900291

[6] Obici S, Zhang BB, Karkanias G et al. Hypothalamic insulin signaling is required for inhibition of glucose production. Nat Med 2002;8:1376–82. 12426561 10.1038/nm1202-798

[7] Tups A, Benzler J, Sergi D et al. Central regulation of glucose homeostasis. Compr Physiol 2017;7:741–64. 28333388 10.1002/cphy.c160015

[8] Niswender KD, Morrison CD, Clegg DJ et al. Insulin activation of phosphatidylinositol 3-kinase in the hypothalamic arcuate nucleus: a key mediator of insulin-induced anorexia. Diabetes 2003;52:227–31. 12540590 10.2337/diabetes.52.2.227

[9] Tups A, Anderson GM, Rizwan M et al. Both p110α and p110β isoforms of phosphatidylinositol 3-OH-kinase are required for insulin signalling in the hypothalamus. J Neuroendocrinol 2010;22:534–42. 20236230 10.1111/j.1365-2826.2010.01975.x

[10] Benzler J, Ganjam GK, Pretz D et al. Central inhibition of IKKβ/NF-κB signaling attenuates high-fat diet-induced obesity and glucose intolerance. Diabetes 2015;64:2015–27. 25626735 10.2337/db14-0093

[11] Zhang X, Zhang G, Zhang H et al. Hypothalamic IKKβ/NF-κB and ER stress link overnutrition to energy imbalance and obesity. Cell 2008;135:61–73. 18854155 10.1016/j.cell.2008.07.043 PMC2586330

[12] Cai D, Liu T. Hypothalamic inflammation: a double-edged sword to nutritional diseases. Ann N Y Acad Sci 2011;1243:E1–39. 22417140 10.1111/j.1749-6632.2011.06388.x PMC4389774

[13] Padmavathi G, Roy NK, Bordoloi D et al. Butein in health and disease: a comprehensive review. Phytomedicine 2017;25:118–27. 28190465 10.1016/j.phymed.2016.12.002

[14] Kim KH, Moon E, Choi SU et al. Identification of cytotoxic and anti-inflammatory constituents from the bark of *Toxicodendron vernicifluum* (Stokes) F.A. Barkley. J Ethnopharmacol 2015;162:231–7. 25582488 10.1016/j.jep.2014.12.071

[15] Price JR. The yellow coloring matter of Dahlia variabilis. J Chem Soc 1939;218:1017–8.

[16] Calligaris SD, Lecanda M, Solis F et al. Mice long-term high-fat diet feeding recapitulates human cardiovascular alterations: an animal model to study the early phases of diabetic cardiomyopathy. PLoS One 2013;8:e60931. 23593350 10.1371/journal.pone.0060931 PMC3623942

[17] Li J, Wu H, Liu Y et al. High fat diet induced obesity model using four strains of mice: Kunming, C57BL/6, BALB/c and ICR. Exp Anim 2020;69:326–35. 32188837 10.1538/expanim.19-0148 PMC7445062

[18] Winzell MS, Ahren B. The high-fat diet-fed mouse: a model for studying mechanisms and treatment of impaired glucose tolerance and type 2 diabetes. Diabetes 2004;53:S215–9. 15561913 10.2337/diabetes.53.suppl_3.s215

[19] Obici S, Feng Z, Karkanias G et al. Decreasing hypothalamic insulin receptors causes hyperphagia and insulin resistance in rats. Nat Neurosci 2002;5:566–72. 12021765 10.1038/nn0602-861

[20] Pocai A, Lam TKT, Gutierrez-Juarez R et al. Hypothalamic K_ATP_ channels control hepatic glucose production. Nature 2005;434:1026–31. 15846348 10.1038/nature03439

[21] Gao Y, Bielohuby M, Fleming T et al. Dietary sugars, not lipids, drive hypothalamic inflammation. Mol Metab 2017;6:897–908. 28752053 10.1016/j.molmet.2017.06.008 PMC5518723

[22] Tsaousidou E, Paeger L, Belgardt BF et al. Distinct roles for JNK and IKK activation in agouti-related peptide neurons in the development of obesity and insulin resistance. Cell Rep 2014;9:1495–506. 25456138 10.1016/j.celrep.2014.10.045

[23] Pretz D, Le Foll C, Rizwan MZ et al. Hyperleptinemia as a contributing factor for the impairment of glucose intolerance in obesity. FASEB J 2021;35:e21216. 33230896 10.1096/fj.202001147R

[24] Ishikawa C, Senba M, Mori N. Butein inhibits NF-κB, AP-1 and Akt activation in adult T-cell leukemia/lymphoma. Int J Oncol 2017;51:633–43. 28586006 10.3892/ijo.2017.4026

[25] Pandey MK, Sandur SK, Sung B et al. Butein, a tetrahydroxychalcone, inhibits nuclear factor (NF)-κB and NF-κB-regulated gene expression through direct inhibition of IκBα kinase β on cysteine 179 residue. J Biol Chem 2007;282:17340–50. 17439942 10.1074/jbc.M700890200

[26] Kuri P, Ellwanger K, Kufer TA et al. A high-sensitivity bi-directional reporter to monitor NF-κB activity in cell culture and zebrafish in real time. J Cell Sci 2017;130:648–57. 27980067 10.1242/jcs.196485

[27] Sakai J, Cammarota E, Wright JA et al. Lipopolysaccharide-induced NF-κB nuclear translocation is primarily dependent on MyD88, but TNFα expression requires TRIF and MyD88. Sci Rep 2017;7:1428. 28469251 10.1038/s41598-017-01600-y PMC5431130

[28] Bansal N. Prediabetes diagnosis and treatment: a review. World J Diabetes 2015;6:296–303. 25789110 10.4239/wjd.v6.i2.296 PMC4360422

[29] El Homsi M, Ducroc R, Claustre J et al. Leptin modulates the expression of secreted and membrane-associated mucins in colonic epithelial cells by targeting PKC, PI3K, and MAPK pathways. Am J Physiol Gastrointest Liver Physiol 2007;293:G365–73. 17495032 10.1152/ajpgi.00091.2007

[30] Banks WA. The blood–brain barrier as an endocrine tissue. Nat Rev Endocrinol 2019;15:444–55. 31127254 10.1038/s41574-019-0213-7

[31] Watanabe Y, Nagai Y, Honda H et al. Isoliquiritigenin attenuates adipose tissue inflammation *in vitro* and adipose tissue fibrosis through inhibition of innate immune responses in mice. Sci Rep 2016;6:23097. 26975571 10.1038/srep23097 PMC4791553

[32] Ishibashi R, Furusawa Y, Honda H et al. Isoliquiritigenin attenuates adipose tissue inflammation and metabolic syndrome by modifying gut bacteria composition in mice. Mol Nutr Food Res 2022;66:e2101119. 35297188 10.1002/mnfr.202101119

[33] Song M-Y, Jeong G-S, Kwon K-B et al. Sulfuretin protects against cytokine-induced β-cell damage and prevents streptozotocin-induced diabetes. Exp Mol Med 2010;42:628–38. 20661005 10.3858/emm.2010.42.9.062 PMC2947020

[34] Guo J, Liu A, Cao H et al. Biotransformation of the chemopreventive agent 2’,4’,4-trihydroxychalcone (isoliquiritigenin) by UDP-glucuronosyltransferases. Drug Metab Dispos 2008;36:2104–12. 18653743 10.1124/dmd.108.021857 PMC2615638

[35] Jin MJ, Kim IS, Park JS et al. Pharmacokinetic profile of eight phenolic compounds and their conjugated metabolites after oral administration of *Rhus verniciflua* extracts in rats. J Agric Food Chem 2015;63:5410–6. 25998231 10.1021/acs.jafc.5b01724

[36] Ruud J, Steculorum SM, Bruning JC. Neuronal control of peripheral insulin sensitivity and glucose metabolism. Nat Commun 2017;8:15259. 28469281 10.1038/ncomms15259 PMC5418592

[37] Lundqvist MH, Almby K, Abrahamsson N et al. Is the brain a key player in glucose regulation and development of type 2 diabetes? Front Physiol 2019;10:457. 31133864 10.3389/fphys.2019.00457 PMC6524713

[38] Yoon NA, Diano S. Hypothalamic glucose-sensing mechanisms. Diabetologia 2021;64:985–93. 33544170 10.1007/s00125-021-05395-6 PMC8087998

[39] Thaler JP, Yi C-X, Schur EA et al. Obesity is associated with hypothalamic injury in rodents and humans. J Clin Invest 2012;122:153–62. 22201683 10.1172/JCI59660 PMC3248304

[40] Ballabh P, Braun A, Nedergaard M. The blood-brain barrier: an overview: structure, regulation, and clinical implications. Neurobiol Dis 2004;16:1–13. 15207256 10.1016/j.nbd.2003.12.016

[41] Abd El Mohsen MM, Kuhnle G, Rechner AR et al. Uptake and metabolism of epicatechin and its access to the brain after oral ingestion. Free Radic Biol Med 2002;33:1693–702. 12488137 10.1016/s0891-5849(02)01137-1

[42] Faria A, Mateus N, Calhau C. Flavonoid transport across blood-brain barrier: implication for their direct neuroprotective actions. Nutr Aging 2012;1:89–97.

[43] Peng H, Cheng FC, Huang YT et al. Determination of naringenin and its glucuronide conjugate in rat plasma and brain tissue by high-performance liquid chromatography. J Chromatogr B Biomed Sci Appl 1998;714:369–74. 9766878 10.1016/s0378-4347(98)00204-7

[44] Suganuma M, Okabe S, Oniyama M et al. Wide distribution of [^3^H](-)-epigallocatechin gallate, a cancer preventive tea polyphenol, in mouse tissue. Carcinogenesis 1998;19:1771–6. 9806157 10.1093/carcin/19.10.1771

[45] Abd El Mohsen MM, Kuhnle G, Rechner AR et al. Uptake and metabolism of epicatechin and its access to the brain after oral ingestion. Free Radic Biol Med 2002;33:1693–702. 12488137 10.1016/s0891-5849(02)01137-1

[46] Faria A, Pestana D, Teixeira D et al. Flavonoid transport across RBE4 cells: a blood-brain barrier model. Cell Mol Biol Lett 2010;15:234–41. 20140760 10.2478/s11658-010-0006-4 PMC6275689

[47] Scapagnini G, Vasto S, Abraham NG et al. Modulation of Nrf2/ARE pathway by food polyphenols: a nutritional neuroprotective strategy for cognitive and neurodegenerative disorders. Mol Neurobiol 2011;44:192–201. 21499987 10.1007/s12035-011-8181-5 PMC5554938

[48] Vauzour D, Vafeiadou K, Rice-Evans C et al. Activation of pro-survival Akt and ERK1/2 signalling pathways underlie the anti-apoptotic effects of flavanones in cortical neurons. J Neurochem 2007;103:1355–67. 17961201 10.1111/j.1471-4159.2007.04841.x

[49] AlJabr AM, Hussain A, Rizwan-Ul-Haq M et al. Toxicity of plant secondary metabolites modulating detoxification genes expression for natural red palm weevil pesticide development. Molecules 2017;22:169. 28117698 10.3390/molecules22010169 PMC6155707

[50] Ugwah-Oguejiofor CJ, Okoli CO, Ugwah MO et al. Acute and sub-acute toxicity of aqueous extract of aerial parts of *Caralluma dalzielii* N. E. Brown in mice and rats. Heliyon 2019;5:e01179. 30775575 10.1016/j.heliyon.2019.e01179 PMC6356088

[51] Mehlem A, Hagberg CE, Muhl L et al. Imaging of neutral lipids by oil red O for analyzing the metabolic status in health and disease. Nat Protoc 2013;8:1149–54. 23702831 10.1038/nprot.2013.055

[52] Koch C, Augustine RA, Steger J et al. Leptin rapidly improves glucose homeostasis in obese mice by increasing hypothalamic insulin sensitivity. J Neurosci 2010;30:16180–7. 21123564 10.1523/JNEUROSCI.3202-10.2010 PMC3697436

[53] Kamstra K, Rizwan MZ, Grattan DR et al. Leptin regulates glucose homeostasis via the canonical Wnt pathway in the zebrafish. FASEB J 2022;36:e22207. 35188286 10.1096/fj.202101764R

[54] Nair AB, Jacob S. A simple practice guide for dose conversion between animals and human. J Basic Clin Pharm 2016;7:27–31. 27057123 10.4103/0976-0105.177703 PMC4804402

[55] Du Bois D, Du Bois EF. A formula to estimate the approximate surface area if height and weight be known. 1916. Nutrition 1989;5:303–11; discussion 312–3. 2520314

