# Supplementary material for: A dahlia flower extract has antidiabetic properties by improving insulin function in the brain
Source: Life Metab. 2023 Jun 18;2(4):load026. doi: 10.1093/lifemeta/load026 (PMC11749471; doi:10.1093/lifemeta/load026)
Supplement: load026_suppl_Supplementary_Material [file load026_suppl_Supplementary_Material.docx]

**Supplementary materials for**

**A dahlia flower extract has anti-diabetic properties by improving insulin function in the brain**

Dominik Pretz, Philip M. Heyward, Jeremy Krebs, Joel Gruchot, Charles Barter, Pat Silcock, Nerida Downes, Mohammed Zubair Rizwan, Alisa Boucsein, Julia Bender, Elaine J. Burgess, Geke A Boer, Pramuk Keerthisinghe, Nigel B. Perry, Alexander Tups

**Detailed materials and methods for flavonoid purification, identification, and HPLC analyses**

*Flower extracts and flavonoids*

Flavonoids were purified from fresh yellow dahlia flowers (*Dahlia pinnata*). Fresh petals were macerated in 96% ethanol (EtOH) at room temperature for 18 h, then the extract was filtered and stored at -18°C. A sub-sample of this extract was rotary-evaporated to dryness (35°C) to give a yellow oily gum. This was bound onto C18 (octadecyl-functionalised silica gel, Aldrich) and subjected to reversed phase (RP) chromatography using a prepacked C18 column (Isolute) which had been preconditioned with EtOH, EtOH:H_2_O (1:1), and H_2_O. This column was developed using H_2_O (4 column volumes, 1 cv = 15 mL); 1:9, 1:4, and 3:7 EtOH:H_2_O (2 cv fractions each); 2:3 EtOH:H_2_O (6 cv fractions); 1:1 EtOH:H_2_O and EtOH (2 cv fractions each). Flavonoid-rich orange fractions eluted with 2:3 EtOH:H_2_O were subjected to preparative HPLC (see below). Sulfuretin, luteolin, butein, apigenin, diosmetin, isoliquiritigenin, and 4-O-methylbutein were identified by comparing ^1^H NMR spectra with literature data (Supplementary Table S1) and by 2D NMR when needed.

Flavonoid glycosides were purified for identification from freeze-dried yellow dahlia petals. Dried petals were finely ground and extracted overnight in 96% EtOH, then the extract was filtered (sintered funnel, porosity no 1) and evaporated to dryness (35 °C) to give a yellow oily gum. This was bound onto C18 and subjected to RP chromatography on a prepacked C18 column preconditioned as above. This column was developed as above, except that 1 cv = 10 mL. Fractions 7 and 8 (30 mg) eluted with 3:7 EtOH:H_2_O were subjected to preparative HPLC (see below). Butein 4’-glucoside and isoliquiritigenin 4’-glucoside (Supplementary Fig. S3) were identified by comparing ^1^H NMR spectra with literature data (Supplementary Table S1) and by 2D NMR.

The extract used in the mouse trials was also prepared from fresh dahlia flowers grown in Southland (New Zealand). ^1^H NMR showed that glucose was the main component in this extract. HPLC analyses (see below) showed the flavonoid compositions (Supplementary Table S2).

For mouse trials of pure flavonoids and mixtures, commercial samples of butein (Sigma-Aldrich, purity 98%) and isoliquiritigenin (AK Scientific 97%) were used. Sulfuretin was purified from dahlia extract as above (purity >98% by HPLC and ^1^H NMR analyses).

The extract used in the human trial was prepared as follows. Fresh petals were removed from flowerheads and freeze-dried, then extracted with 1:1 w/w EtOH:H_2_O (previously sparged with O_2_-free N_2_), by maceration in the dark at 20−25°C for 48 h. The petals were then removed from the extract, which was rotary evaporated to remove most of the EtOH, then blended with microcrystalline cellulose (MCC). The resulting slurry was freeze-dried to produce a slightly sticky friable yellow powder. The powder was packed into Vege-caps at different dose levels based on total extract weight per capsule (Supplementary Table S2). Different doses were made by further blending the powder-extract mix with more MCC. The capsules were hand-packed using the Cap-M-Quik filler machine (Empty Caps Company, Ca, USA). Capsules were then stored at -20°C in sterile plastic containers flushed with O_2_-free N_2_, in vacuum-sealed foil laminate bags. HPLC analyses (see below) showed the yellow flavonoid compositions given in Supplementary Table S2.

*HPLC analysis*

Analytical reversed phase HPLC was carried out using an Agilent HP1100 instrument, controlled with Agilent OpenLab software, at 20°C on a C18 column (Phenomenex Luna ODS(3) 5 μm 100 Å 150 x 3 mm) with a 2 x 4 mm C18 guard column. The sample solution injection volume was 5 µL, mobile phase flow rate was 0.5 mL/min of MeCN and H_2_O, both with 0.1% formic acid. The solvent program was linear, from 10% MeCN to 100% MeCN over 12.5 min, 2.5 min hold, then back to 10% MeCN over 1 min, 4 min hold before the next injection. Peaks were detected at 206 nm, 262 nm, 348 nm, and 382 nm, and UV spectra were recorded from 190 nm to 600 nm using a diode array detector. Quantitative analyses (Supplementary Table S2) used 382 nm detection with response factors (Supplementary Table S1) determined by injections of purified compounds at several concentrations.

**
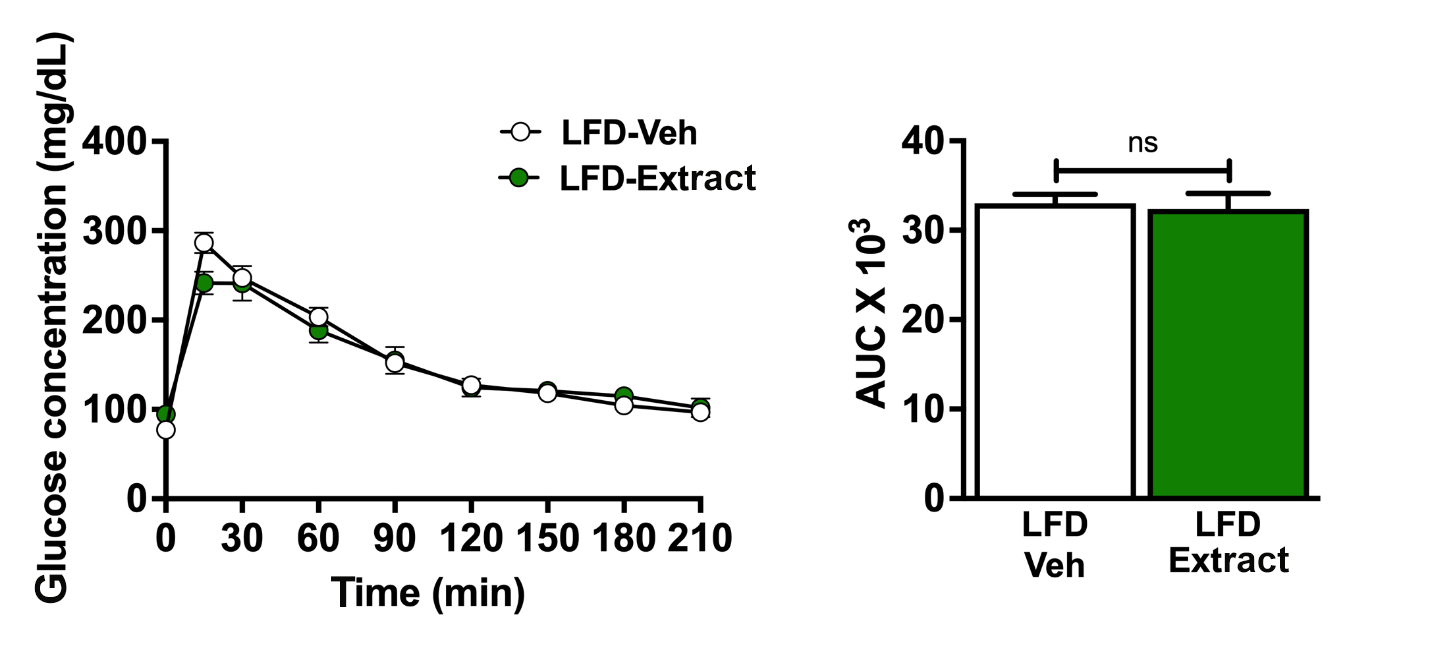
**

**Supplementary Figure S1 The dahlia extract did not lead to hypoglycemia in healthy mice.** C57BL/6 mice were fed the LFD for 4 weeks and treated with the extract orally (10 mg/kg) 1 h before an intraperitoneal glucose tolerance test (ipGTT). ipGTT blood glucose response curve (left panel) and corresponding AUC (right panel; *n* = 6). Data are represented as the mean ± SEM.

| **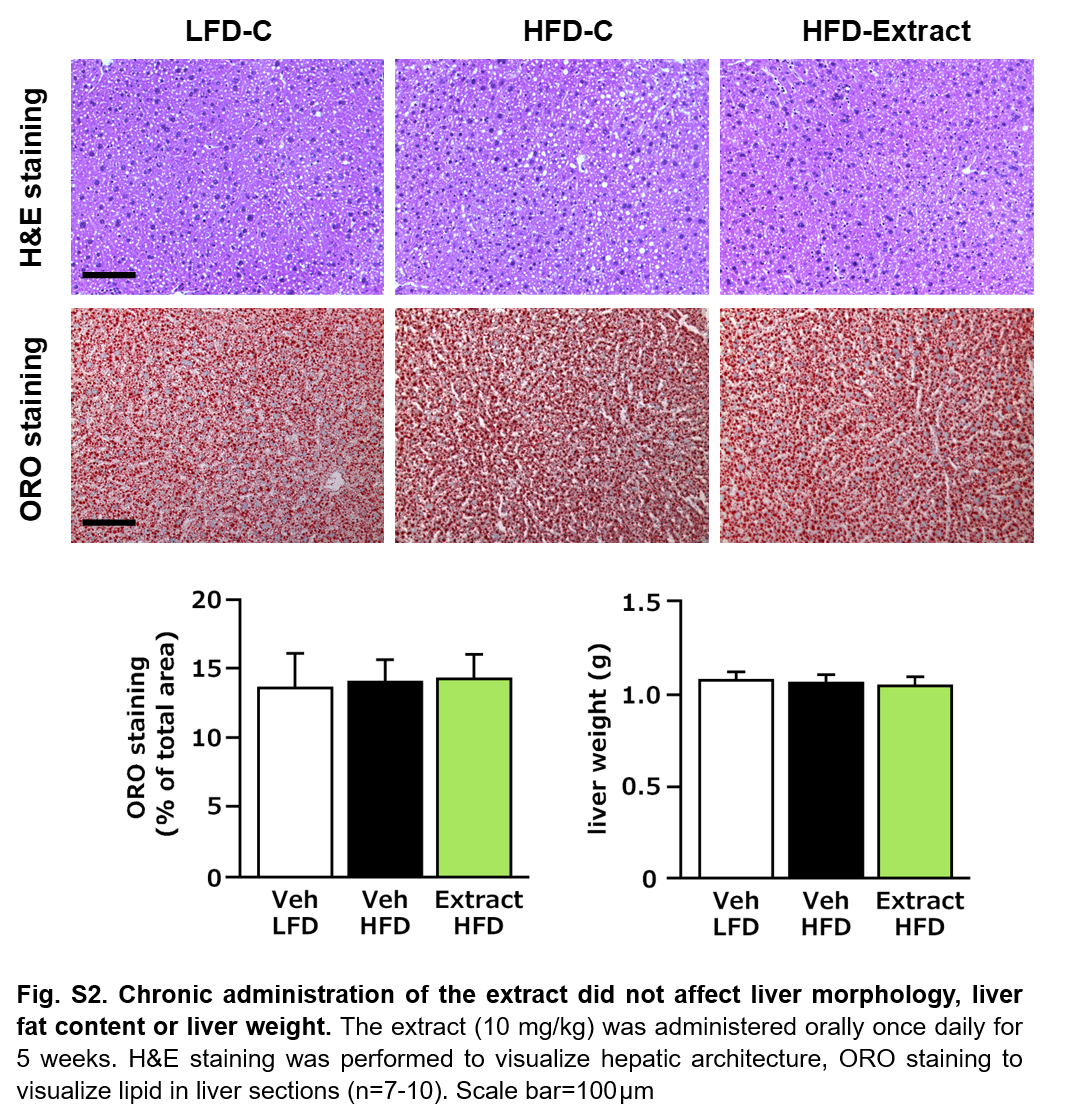** |
| --- |
| **Supplementary Figure S2** **Liver morphology and lipids after long-term extract treatment.** The extract (10 mg/kg) was administered orally once daily for 5 weeks. H&E staining was performed to visualize hepatic architecture, and ORO staining was performed to visualize lipids in liver sections (*n* = 7−10). Scale bar = 100 μm. |

| **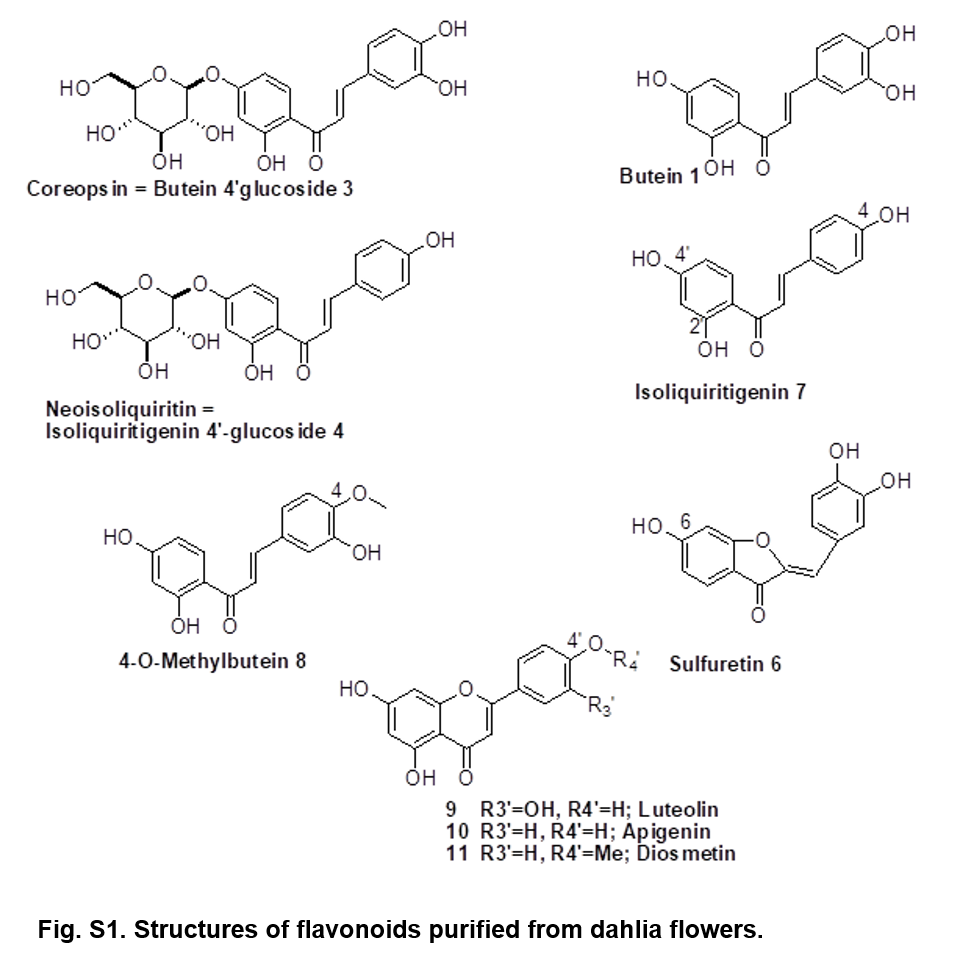** |
| --- |
| **Supplementary Figure S3 Structures of flavonoids purified from dahlia flowers.** |

| **Supplementary Table S1 HPLC retention times, UV/Vis response factors, and references for flavonoids purified from dahlia flowers.** |
| --- |
| 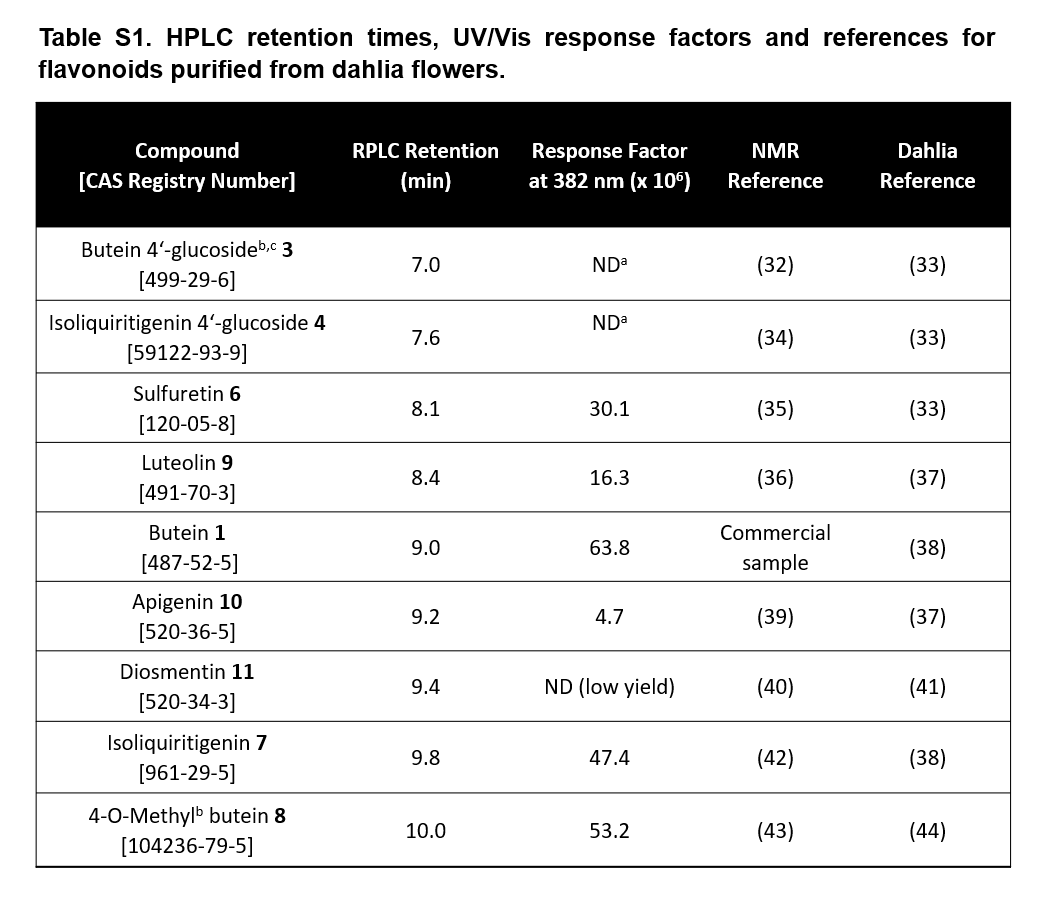 |

ND = Not detected. ^b^Structures in Supplementary Fig S2.

**References for Supplementary Table S1**

32. T. C. Lima, R. J. Souza, A. D. Santos, M. H. Moraes, N. E. Biondo, A. Barison, M. Steindel, M. W. Biavatti, Evaluation of leishmanicidal and trypanocidal activities of phenolic compounds from Calea uniflora Less. Natural product research 30, 551-557 (2016).

33. C. Nordström, T. Swain, The flavonoid glycosides of Dahlia variabilis. II. Glycosides of yellow varieties “Pius IX” and “Coton”. Archives of Biochemistry and Biophysics 60, 329-344 (1956).

34. J. E. Lee, J. Y. Lee, J. Kim, K. Lee, S. U. Choi, S. Y. Ryu, Two minor chalcone acetylglycosides from the roots extract of Glycyrrhiza uralensis. Archives of pharmacal research 38, 1299-1303 (2015).

35. G. M. V. Junior, C. M. de M. Sousa, A. J. Cavalheiro, J. H. G. Lago, M. H. Chaves, Phenolic derivatives from fruits of Dipteryx lacunifera Ducke and evaluation of their antiradical activities. Helvetica Chimica Acta 91, 2159-2167 (2008).

36. Y. Park, B. H. Moon, E. Lee, Y. Lee, Y. Yoon, J. H. Ahn, Y. Lim, 1H and 13C‐NMR data of hydroxyflavone derivatives. Magnetic Resonance in Chemistry 45, 674-679 (2007).

37. T. Nakaoki, Flavone coloring matter. V. Constituents of the flowers of Dahlia variabilis, Desf. Diosmin. Yakugaku Zasshi 58, 639-647 (1938).

38. S. T. Bate-Smith EC, The isolation of 2, 4, 4′-trihydroxychalcone from yellow varieties of Dahlia variabilis. J Chem Soc, 2185–2187 (1953).

39. M. Miyazawa, M. Hisama, Antimutagenic activity of flavonoids from Chrysanthemum morifolium. Bioscience, biotechnology, and biochemistry 67, 2091-2099 (2003).

40. B. N. Timmermann, R. Mues, T. J. Mabry, A. M. Powell, 6-methoxyflavonoids from Brickellia laciniata (compositae). Phytochemistry 18, 1855-1858 (1979).

41. D. E. Giannasi, Flavonoid chemistry and evolution in Dahlia (Compositae). Bulletin of the Torrey Botanical Club, 404-412 (1975).

42. M. Namikoshi, H. Nakata, M. Nuno, T. Ozawa, T. Saitoh, Homoisoflavonoids and related compounds. III. Phenolic constituents of Caesalpinia japonica Sieb. et Zucc. Chemical and pharmaceutical bulletin 35, 3568-3575 (1987).

43. E. Maldonado, M. Bello, J. L. Villaseñor, A. Ortega, Acyclic diterpenes from Perymenium hintonii. Phytochemistry 49, 1115-1118 (1998).

44. J. Lam, P. Wrang, Flavonoids and polyacetylenes in Dahlia tenuicaulis. Phytochemistry 14, 1621-1623 (1975).

| **Supplementary Table S2 Flavonoid contents of fresh dahlia flower extract used in mouse trials and those of capsules used in human clinical trial.** |
| --- |
| **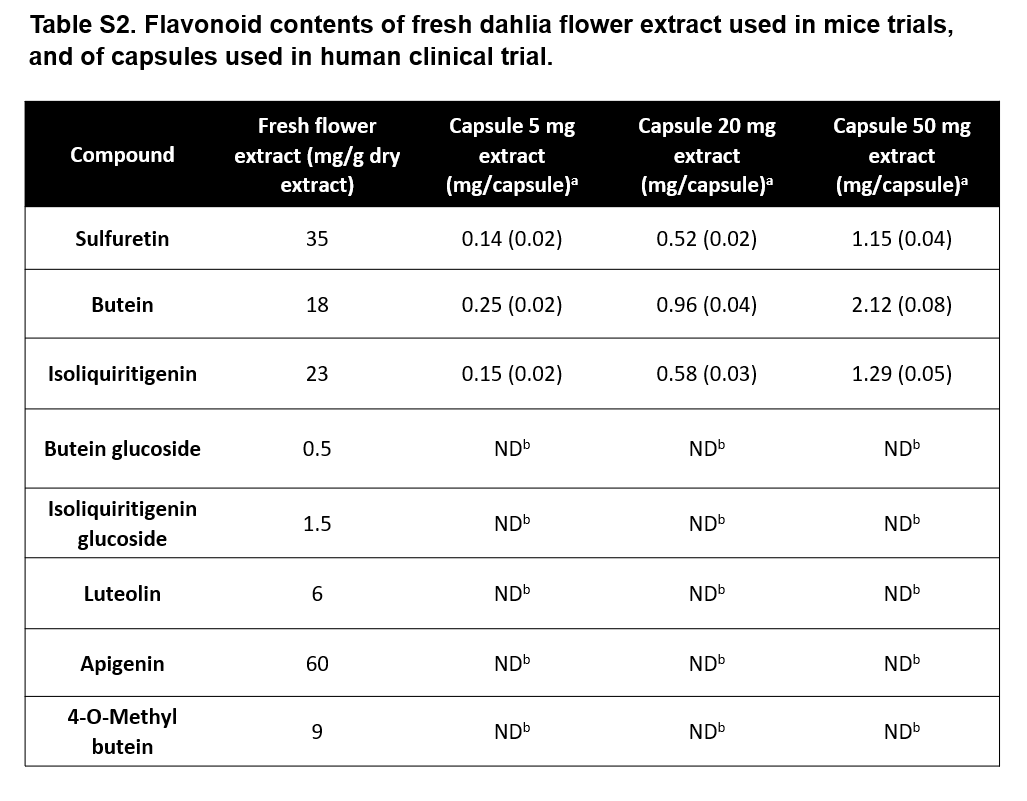** |

^a^Mean (Standard Deviation) for three analyses. ^b^ND = Not detected.

|  |
| --- |
|  |
